# Bispezifische Antikörper in der Therapie des Prostatakarzinoms

**DOI:** 10.1007/s00120-025-02574-w

**Published:** 2025-04-02

**Authors:** Susanne Jung, Jonas Heitmann, Martin Pflügler, Gundram Jung, Steffen Rausch, Helmut Salih

**Affiliations:** KKE Translationale Immunologie, Universitätsklinikum Tübingen, Otfried-Müller-Str. 10, 72076 Tübingen, Deutschland

**Keywords:** PSMA, Immuntherapie, T-Zell-basiert, Biochemisches Rezidiv, Klinische Studien, PSMA, Immunotherapy, T cell based, Biochemical relapse, Clinical trials

## Abstract

Das Prostatakarzinom (PC) ist die zweithäufigste Krebserkrankung bei Männern. Sobald die Krankheit auf eine Androgenentzugstherapie nicht mehr anspricht, sind die verbleibenden Behandlungsoptionen begrenzt. Trotz intensiver Bemühungen konnte bislang nur wenige der T‑Zell-basierten immuntherapeutischen Strategien, die mittlerweile bei der Behandlung anderer Krebserkrankungen etabliert sind, für das PC erfolgreich implementiert werden. Dies gilt sowohl für die Immun-Checkpoint-Inhibition, welche die T‑Zell-Immunität generell verstärkt, jedoch bislang keine überzeugenden Ergebnisse erbracht hat, als auch für chimäre Antigenrezeptor‑T (CART)-Zellen und bispezifische Antikörper (bsAbs), die T‑Zellen gezielt gegen Tumorzellen mobilisieren. Im Vergleich zu CART-Zellen bieten bsAbs den Vorteil, dass sie als „Off the shelf“-Reagenzien unmittelbar verfügbar sind, was eine Therapieverzögerung vermeidet und mit geringerem Kostenaufwand verbunden ist. Aktuell befinden sich mehrere bsAbs zur Behandlung des PC in Entwicklung. Während einige Präparate aufgrund von Nebenwirkungen und Anti-drug-antibody-Bildung mittlerweile nicht mehr weiterverfolgt werden, haben andere vielversprechende erste Ergebnisse geliefert. Hierzu gehören insbesondere gegen STEAP1 und gegen PSMA gerichtete bsAbs, die mittlerweile neben der metastasierten Situation auch im biochemischen Rezidiv evaluiert werden. Die zugrundeliegenden Konzepte sowie der aktuelle Stand der klinischen Entwicklung, sowie die zukünftigen Perspektiven werden dargestellt und diskutiert.

## Abstract (en)

Prostate cancer (PC) is the second most common cancer in men. As soon as androgen deprivation therapy fails, treatment options are limited. Despite intense efforts, hardly any of the T cell-based immunotherapeutic strategies that have revolutionized oncological treatment in other cancer entities are yet established for the treatment of PC. This includes immune checkpoint inhibition, which generally reinforces T cell-immunity but failed to achieve broad activity in PC, as well as chimeric antigen receptor T (CART) cells and bispecific antibodies (bsAbs), which specifically mobilize T cells against tumor cells. Compared to CART cells, bsAbs have the advantage of being readily available “off-the-shelf” reagents. Currently several bispecific constructs are in development for PC. While development of some was discontinued due to substantial side effects or development of anti-drug antibodies, others have yielded promising results. These include in particular bsAbs directed against six-transmembrane epithelial antigen of the prostate 1 (STEAP1) and prostate-specific membrane antigen (PSMA), which are currently being evaluated in both patients with metastasized disease and biochemical relapse. The concepts underlying the different constructs, the current status of clinical development, and future perspectives are discussed.

Das Prostatakarzinom (PC) ist die zweithäufigste Krebserkrankung bei Männern. Immuntherapien, die bei anderen Krebserkrankungen eine wichtige Therapieoption darstellen, sind beim PC bislang nicht etabliert. Dies gilt auch für bispezifische Antikörper (bsAbs), über die nach Bindung an Tumorzellen T‑Zellen stimuliert werden. Bei hämatologischen Neoplasien sind diese therapeutisch sehr erfolgreich, auch beim PC werden sie mit vielversprechenden Ergebnissen erprobt. Verschiedene in Entwicklung befindliche bsAbs werden hinsichtlich zugrundeliegender Konzepte und Perspektiven zur Behandlung des PC diskutiert.

Das PC ist weltweit die zweithäufigste Krebserkrankung bei Männern, mit etwa 1,5 Mio. neu diagnostizierten Fällen und 397.000 Todesfällen im Jahr 2022 [2]. Die aktuelle Erstlinientherapie des metastasierten hormonsensitiven PC (mHSPC) umfasst in der Regel eine Kombination aus Androgendeprivationstherapie (ADT) und zusätzlichen Therapieoptionen. Diese können aus Substanzen wie Docetaxel, Darolutamid, Abirateron, Enzalutamid oder Apalutamid bestehen, abhängig von individuellen Patientenmerkmalen und Krankheitsausbreitung [5]. Jedoch kommt es unter Therapie häufig zur Entwicklung einer Kastrationsresistenz [18]. Die Behandlung von Patienten mit metastasiertem kastrationsresistentem PC (mCRPC) mit den Zytostatika Docetaxel und Cabazitaxel erreicht einen medianen Gesamtüberlebens-(OS)-Vorteil von bis zu 19 Monaten [6]. Ähnliche Ergebnisse zeigen sich in der kastrationsresistenten Situation für die Radioligandentherapie mit ^177^Lutetium-PSMA [12]. Auch Poly(ADP-Ribose) Polymerase (PARP)-Inhibitoren sind mittlerweile für das mCRPC zugelassen, kommen allerdings nicht für jeden Patienten in Frage [4].

In vielen Tumorentitäten hat in den letzten Jahren die Einführung der Immuntherapie die Behandlung revolutioniert [15]. Besonders solche Strategien, die T‑Zellen als zentrale Komponenten des adaptiven Immunsystems gegen Tumorzellen mobilisieren, haben große Fortschritte erzielt. Physiologisch wird die Aktivierung von T‑Zellen durch zwei wesentliche Signale reguliert: „Signal 1“ erfolgt über den antigenspezifischen T‑Zell-Rezeptor/CD3-Komplex (TCR/CD3), der Peptide erkennt, die durch MHC-Moleküle präsentiert werden. „Zweitsignale“ über kostimulatorische und/oder koinhibitorische („Immune checkpoint“-)Rezeptoren bestimmen, ob eine effektive und langanhaltende Immunantwort erfolgt [8]. Zugelassene Ansätze der T‑Zell-basierten Krebstherapie umfassen die sog. Immun-Checkpoint-Inhibitoren (ICI), chimäre Antigenrezeptor-T-Zellen (CAR-T-Zellen) und bispezifische Antikörper (bsAb). Letztere stehen im Mittelpunkt dieser Arbeit.

Beim PC werden immuntherapeutische Strategien teilweise eingesetzt, wie z. B. das in den USA zugelassene Sipuleucel‑T [21], andere, wie RNA-basierte Vakzinierungen [22] oder ICI wurden intensiv klinisch getestet, bislang jedoch ohne Ergebnisse, die eine umfassende klinische Implementierung rechtfertigen [17]. Allenfalls konnte die CONTACT-02-Studie, welche die Wirksamkeit des Tyrosinkinase-Inhibitors Cabozantinib in Kombination mit ICI untersuchte, einen positiven Effekt auf das progressionsfreien Überleben zeigen [1].

Im Gegensatz zu ICI, deren Wirkprinzip auf einer generellen bzw. unspezifischen Verstärkung der T‑Zell-Immunität durch Aufhebung hemmender Signale beruht, bewirken CART-Zellen und bsAbs durch Verwendung von Antikörpern gegen tumorassoziierte Antigene eine zielgerichtete Antitumorimmunität. BsAbs, die nach Bindung an ihr Zielantigen auf Tumorzellen T‑Zellen aktivieren, ebenso wie die ihnen eng verwandten CART-Zellen (gentechnisch modifizierte T‑Zellen des individuellen Patienten), zielen häufig auf dieselben Zielantigene ab. BsAbs sind jedoch – im Vergleich zu CART-Zellen – standardisierte „Off the shelf“-Reagenzien, deren Aktivität pharmakokinetisch kontrollierbar ist, wodurch diese ein sehr attraktives Therapiekonzept darstellen. Nachfolgend werden verschiedene derzeit zur Behandlung des PC in Entwicklung befindliche bsAbs hinsichtlich der zugrundeliegenden Konzepte und Perspektiven diskutiert.

## Hintergrund

**Abb. 1:**
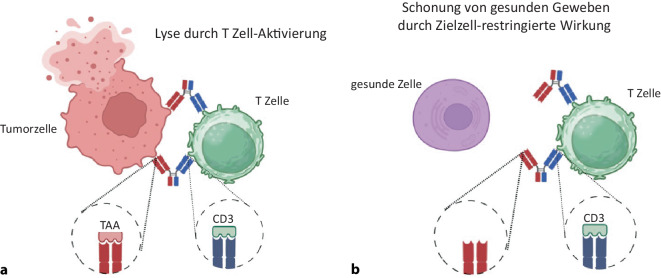
Zielgerichtete T‑Zell-Aktivierung mit bispezifischen Antikörpern (bsAbs). **a** Mobilisierung von T‑Zellen gegen Tumorzellen durch bsAbs mit Spezifität für tumorassoziierte Antigene und CD3. **b** Keine Expression des Zielantigens auf gesunden Zellen, dadurch keine „Off-target“-Aktivierung in Abwesenheit von Tumorzellen

Antikörper kamen erstmals in Form von humanisierten monoklonalen Antikörpern (mAbs) in der Krebstherapie zum Einsatz. Ihre therapeutische Wirkung beruht wesentlich auf der Stimulation Fc-Rezeptor-tragender Immuneffektorzellen, was zur Lyse der Tumorzellen insbesondere durch Natürliche Killer (NK)-Zellen führt. Eine Stimulation von T‑Zellen ist mit mAbs jedoch nicht möglich, da T‑Zellen in der Regel keine Fc-Rezeptoren exprimieren. Eine Rekrutierung von T‑Zellen kann jedoch mit bsAbs erreicht werden, die mit ihrem Ziel-Arm an ein tumorassoziiertes Antigen (TAA) binden und mit ihrem Effektorarm üblicherweise durch Aktivierung des TCR/CD3-Komplex-T-Zellen stimulieren (Abb. 1a). Mittlerweile wurde eine Vielzahl verschiedener bsAb-Konstrukte entwickelt, die sich in zentralen Charakteristika, also hinsichtlich ihrer Formate, Zielantigenbinder und Effektorkomponenten voneinander unterscheiden [3]. Von wesentlicher Bedeutung ist es in jedem Fall, eine unspezifische Aktivierung des T‑Zell-Systems durch fehlende Zielzellrestriktion zu vermeiden, d. h. ungerichtete T‑Zell-Aktivierung, ohne dass der bsAb an eine Tumorzelle gebunden ist, auszuschließen. Aber auch eine übermäßige „On-target-off-tumor“-Aktivierung von T‑Zellen, z. B. durch Expression der Zielantigene auf normalem Gewebe, kann zu lebensbedrohlichem Zytokin-Release-Syndrom (CRS) führen und auch in weniger ausgeprägten Fällen die Applikation höherer Dosierungen mit optimaler antitumoraler Wirksamkeit verhindern. Vor diesem Hintergrund sollte nicht nur das Zielantigen möglichst wenig auf gesunden Zellen exprimiert werden (Abb. 1b); es muss auch die Bindung des bsAbs an Fc-Rezeptor-tragende Zellen vermieden werden, da dies auch in Abwesenheit von Tumorzellen zur Aktivierung von T‑Zellen führen kann.

## Zielantigene von bsAbs im PC

Das bekannteste und am häufigsten als Zielantigen genutzte TAA im PC ist das prostataspezifische Membranantigen (PSMA; [20]). PSMA wird relativ spezifisch auf gesunden und malignen Prostatazellen exprimiert, darüber hinaus auch auf der Neovaskularisation einer Vielzahl verschiedener solider Tumoren, einschließlich beim PC [14]. Die neovaskuläre Expression könnte von großer Bedeutung für den therapeutischen Erfolg sein: bsAbs (und auch CART-Zellen) sind bei soliden Tumoren bislang weit weniger erfolgreich als in hämatologischen Malignomen, was wesentlich auf einen unzureichenden Einstrom der Immunzellen in den Tumor zurückzuführen sein könnte. Dieses Problem könnte durch Zielantigene gelöst werden, die nicht nur auf den Tumorzellen selbst, sondern auch auf Tumorgefäßen exprimiert werden. Ein dadurch mögliches „duales Targeting“ beider Strukturen könnte die Einwanderung von Immunzellen über das geschädigte Endothel mit nachfolgender Zerstörung der Tumorzellen ermöglichen.

Weitere TAA für bsAbs bei PC sind das Prostatastammzellantigen PSCA [10], CD155 [30], Her2/neu [27], ADAM17 (A-Disintegrin und Metalloproteinase 17) [28] sowie kürzlich das „six-transmembrane epithelial antigen of the prostate 1“ (STEAP1; [16]). Insgesamt ist PSMA das am meisten verwendete TAA bei PC. Dies wird auch durch seine klinische Verwendung für die Bildgebung mittels radioaktiver PSMA-Tracer wie ^68^Ga- und ^18^F‑markierten Verbindungen sowie für die Radioligandentherapie mit ^177^Lu-PSMA verdeutlicht [24].

## BsAbs in klinischer Prüfung

Viele verschiedene bsAb-Konstrukte wurden für die PC-Therapie entwickelt, kamen jedoch nicht über präklinische Untersuchungen hinaus. Auf eine Darstellung dieser Konstrukte wird zugunsten der praktischen Relevanz verzichtet und der Fokus auf in klinischer Entwicklung befindliche bsAbs gelegt.

**Abb. 2:**
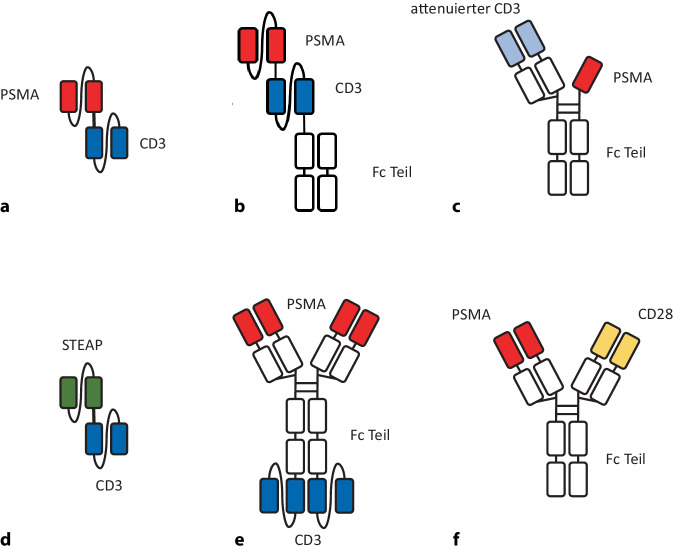
Beispiele für bispezifische Antikörperformate: **a** Pasotuximab/ BAY2010112/ AMG212, ein einkettiges Antikörperfragment im BiTE-Format. **b** AMG160, ein HLE-BiTE mit an Fc-Teil eines IgG Antikörpers fusionierten scFv-Fragmenten. **c** AMG340 mit modifizierter CD3 Bindungsdomäne. **d** AMG509, ein gegen STEAP1 gerichteter bsAb. **e** CC-1 als Beispile des IgGsc Formats. **f** REGN5678, ein gegen PSMA und CD28 gerichteter bsAb

*Pasotuxizumab/BAY 2010112/AMG 212* ist ein bsAb im Format eines sog. „bispecific T cell engager“ (BiTE), bei dem die variablen Domänen zweier Antikörper miteinander verbunden sind (Abb. 2a). Diese Struktur geht mit entsprechend kurzer Halbwertszeit einher. Der Antikörper wurde in einer Phase-I-Studie bei Patienten mit mCRPC getestet. Die maximal verträgliche Dosis konnte aufgrund eines vorzeitigen Studienabbruchs nicht definiert werden, die Entwicklung des Präparats wurde daher abgebrochen [13].

*AMG-160* ist ein BiTE-Molekül der „nächsten Generation“, ein sog. HLE-BiTE, bei dem die beiden scFv-Fragmente des BiTE-Moleküls an den Fc-Teil eines IgG-Antikörpers fusioniert sind (Abb. 2b). Dies führt zu einer verlängerten Halbwertszeit. In einer Phase-I-Studie zur Evaluation dieses Konstrukts wurde ebenfalls keine maximal tolerierte Dosisstufe definiert, ein signifikanter Anteil der Patienten entwickelte hohe Konzentrationen von „anti-drug-antibodies“ gegen das Präparat [7].

Bei der Entwicklung des Nachfolgeprodukts *AMG-340* wurde die CD3-Bindungsdomäne modifiziert, um über eine niedrigere Affinität das Auftreten von CRS zu reduzieren (Abb. 2c). Eine Phase-I-Studie zeigte jedoch keine ausreichende klinische Aktivität, woraufhin die Entwicklung des Präparats ebenfalls eingestellt wurde [9].

*AMG-509 (Xaluritamig)* ist ein gegen STEAP1 gerichteter bsAb mit zwei Anti-STEAP1- und einer Anti-CD3-Bindungsstelle (Abb. 2d). STEAP1 wird bei ca. 80 % aller metastasierten PC exprimiert und ist mit einer schlechten Prognose assoziiert. Aktuell wird der bsAb in einer klinischen Phase-II-Studie evaluiert, nachdem sich in der Phase-I-Studie sehr gute Ansprechraten gezeigt hatten. Die beschriebenen Nebenwirkungen waren mit Muskelschmerzen als wesentlicher Toxizität nicht unerheblich, CRS als die klassische Nebenwirkung eines bsAbs wurde berichtet, schränkte aber die Verträglichkeit kaum ein [16].

An unserer Einrichtung wurde der *PSMAxCD3 bsAb CC‑1* im sog. IgGsc-Format entwickelt [29]. Bei diesem Format sind zwei scFv-Fragmente kovalent an das carboxyterminale Ende des Fc-Teils eines IgG 1-Antikörpers gebunden (Abb. 2e). In einer Phase-I-Studie in Patienten mit metastasiertem PC wurde die Zieldosis ohne Anzeichen einer dosislimitierenden Toxizität erreicht; das in 79 % als häufigste Nebenwirkung auftretende CRS überschritt bei keinem Patienten Grad II. Mit einer Ausnahme zeigten alle 20 der schwer vorbehandelten Patienten ein Ansprechen auf die Zieldosis im Sinne eines schnellen und deutlichen PSA-Abfalls, der bis zu 60 % erreichte [11].

**Abb. 3:**
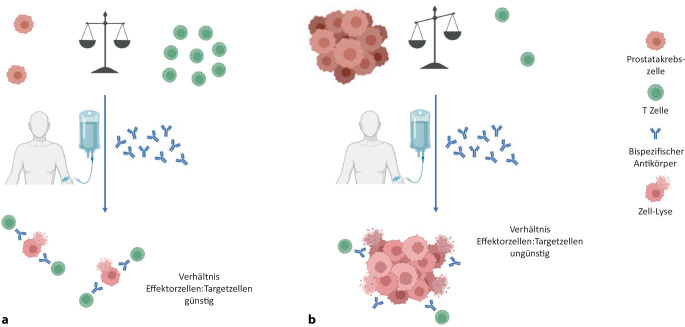
Verhältnis von Effektorzellen (T-Zellen) und Targetzellen (Tumorzellen) in Abhängigkeit vom Tumorstadium. **a** Wenig Tumormasse, z.B. biochemisches Rezidiv. **b** hohe Tumormasse, fortgeschrittene Erkrankungssituation

Aktuell wird CC‑1 in einer weiteren Studie als Erstlinientherapie bei Patienten mit biochemischem Rezidiv evaluiert. In dieser Situation zeigt der ansteigende PSA-Wert ein drohendes Rezidiv an. Die Tumorzellmanifestationen sind jedoch noch so klein, dass sie sich einer (PSMA-PET-) Bildgebung entziehen. Die derzeitige Standardbehandlung in einer solchen Situation ist die Salvage-Bestrahlung (RT) oder Salvage-Prostatektomie (RP). Dies wird gegebenenfalls durch eine längerfristige Hormonbehandlung ergänzt. Diese Therapien bergen ein erhebliches Risiko für Langzeittoxizitäten, mit Harninkontinenz und erektiler Dysfunktion sowie im Falle der RT neben urogenitaler auch rektale Toxizität. Schlägt die lokale Salvage-Therapie fehl, stellt die palliative ADT den Behandlungsstandard dar [5]. In der Hochrisikosituation einer schnellen PSA-Dynamik führten die Ergebnisse der EMBARK-Studie außerdem zur Zulassung von Enzalutamid, allein oder in Kombination mit ADT [25]. Auch außerhalb der Hochrisikosituation besteht jedoch bei Patienten mit biochemischem Rezidiv, im Vergleich zu Patienten mit manifestem metastasiertem PC, eine besonders vorteilhafte Ausgangssituation für eine Immuntherapie. Dies ist darin begründet, dass das Verhältnis von Immunzellen zu Tumorzellen hier deutlich günstiger ist als in fortgeschrittenen Stadien. Zudem ist das Immunsystem nicht durch vorangegangene Therapien kompromittiert, sodass durch eine zeitlich begrenzte bsAb-Therapie ein Therapieerfolg erwartet werden kann (Abb. 3).

Das Verhältnis von Immun- zu Tumorzellen ist im biochemischen Rezidiv optimal für den Erfolg einer Immuntherapie mit bsAbs. Während die oben diskutierten bsAbs eine T‑Zell-Aktivierung durch ein „Signal 1“ über eine Stimulation von CD3 erreichen, sind aktuell alternative Konstrukte, die kostimulatorische Molelüle aktivieren („Signal 2“), in Entwicklung. Hierzu gehört auch *REGN5678*, ein PSMAxCD28 bsAb (Abb. 2f), der in Kombination mit dem PD-1-blockierenden Antikörper Cemiplimab in Patienten mit metastasiertem und/oder kastrationsresistentem PC appliziert wurde. Vorläufige Daten zeigen sowohl bildgebend als auch im PSA-Verlauf Hinweise auf klinische Aktivität, bei jedoch erheblicher Toxizität [26].

In unserer Einrichtung arbeiten wir ebenfalls an bispezifischen Kostimulatoren, die in Kombination mit gegen CD3 gerichteten bsAbs evaluiert werden sollen. Die erste klinische Anwendung ist für 2025 geplant.

## Schlussfolgerungen und Zukunftsperspektiven

Bisher beschränken sich die Erfolge der Immuntherapie mit bsAbs (ebenso wie bei CART-Zellen) v. a. auf hämatologische Malignome. Eine wesentliche Limitation bei soliden Tumoren besteht in der häufig unzureichenden Zugänglichkeit des Tumors für Immuneffektorzellen [23]. Für die Behandlung des PC bietet sich PSMA als gut etabliertes Zielantigen an. Es wird daher bei den meisten in Entwicklung befindlichen T‑Zell-rekrutierenden Strategien eingesetzt. PSMA zeigt ein stark tumorrestringiertes Expressionsmuster, was mit einer Verringerung der Schädigung von gesundem Gewebe assoziiert ist. Ebenso wird aber auch die dosislimitierende „On-target-off-tumor“-T-Zell-Aktivierung reduziert. Zusätzlich ist PSMA auch auf Tumorgefäßen exprimiert [14], was auf einen dualen Wirkmechanismus durch zusätzliche Schädigung der Tumorgefäße und eine Verbesserung des Zugangs von Immunzellen in den Tumor hoffen lässt.

Einen weiteren vielversprechenden Ansatz stellen bsAbs gegen STEAP1 dar. Für diese wurden kürzlich in der metastasierten Situation überzeugende Effizienzdaten berichtet. Eine Überexpression von STEAP1 wurde hauptsächlich in metastasierten, fortgeschrittenen Erkrankungen nachgewiesen, wo eine Korrelation zwischen STEAP1-Expression und Gleason Score bzw. Prognose der Erkrankung gezeigt wurde [19]. Daten zu früheren Erkrankungsstadien stehen aktuell noch aus.

Die Kombination zweier funktionell voneinander abhängiger bsAbs, die gegen zwei Zielantigene, welche beide auf Tumorzellen, nicht jedoch gemeinsam auf gesunden Geweben exprimiert werden und die sowohl CD3 als auch das kostimulatorische Molekül CD28 aktivieren, stellt eine vielversprechende Option dar. Dies ist insbesondere für Patienten mit hoher Tumorlast der Fall.

Insgesamt besteht eine hohe Aktivität bei der Entwicklung von bsAbs zur Behandlung des PC. Neue Konstrukte mit vorteilhaften Eigenschaften und kombinatorische Ansätze versprechen eine Überwindung der aktuellen Einschränkungen. Insbesondere die frühe Erkrankungssituation des biochemischen Rezidivs bietet für immuntherapeutische Ansätze eine vielversprechende Situation für maximale Wirkungsentfaltung. Es bleibt somit zu hoffen, dass durch den Einsatz von bsAbs in den kommenden Jahren ein Durchbruch erzielt werden kann, sodass auch Patienten mit PC von einer Immuntherapie profitieren können. Die derzeitige Entwicklungsaktivität und vorliegende Ergebnisse mit bsAbs im PC lassen auf einen baldigen Durchbruch hoffen.

## Fazit für die Praxis

- Die bsAbs stellen einen neuen, zielgerichteten Ansatz zur Behandlung des PC dar, der im Vergleich zu bislang etablierten Methoden, die in vielen Fällen noch keine zufriedenstellende Krankheitskontrolle ermöglichen, einen Durchbruch verspricht.
- Mehrere verschiedene bsAbs werden aktuell in unterschiedlichen Krankheitsstadien in klinischen Studien überprüft und zeigen erste vielversprechende Ergebnisse.
